# Computational screening for new neuroprotective ingredients against Alzheimer's disease from bilberry by cheminformatics approaches

**DOI:** 10.3389/fnut.2022.1061552

**Published:** 2022-12-09

**Authors:** Ran Xiao, Rui Liang, Yun-hui Cai, Jie Dong, Lin Zhang

**Affiliations:** ^1^Hunan Key Laboratory of Processed Food for Special Medical Purpose, Hunan Key Laboratory of Forestry Edible Resources Safety and Processing, School of Food Science and Engineering, National Engineering Research Center of Rice and Byproduct Deep Processing, Central South University of Forestry and Technology, Changsha, China; ^2^Xiangya School of Pharmaceutical Science, Central South University, Changsha, China; ^3^Sinocare Inc., Changsha, China

**Keywords:** anthocyanins, Alzheimer's disease, cheminformatics, antioxidation, anti-aggregation

## Abstract

Bioactive ingredients from natural products have always been an important resource for the discovery of drugs for Alzheimer's disease (AD). Senile plaques, which are formed with amyloid-beta (Aβ) peptides and excess metal ions, are found in AD brains and have been suggested to play an important role in AD pathogenesis. Here, we attempted to design an effective and smart screening method based on cheminformatics approaches to find new ingredients against AD from *Vaccinium myrtillus* (bilberry) and verified the bioactivity of expected ingredients through experiments. This method integrated advanced artificial intelligence models and target prediction methods to realize the stepwise analysis and filtering of all ingredients. Finally, we obtained the expected new compound malvidin-3-O-galactoside (Ma-3-gal-Cl). The *in vitro* experiments showed that Ma-3-gal-Cl could reduce the OH· generation and intracellular ROS from the Aβ/Cu^2+^/AA mixture and maintain the mitochondrial membrane potential of SH-SY5Y cells. Molecular docking and Western blot results indicated that Ma-3-gal-Cl could reduce the amount of activated caspase-3 *via* binding with unactivated caspase-3 and reduce the expression of phosphorylated p38 *via* binding with mitogen-activated protein kinase kinases-6 (MKK6). Moreover, Ma-3-gal-Cl could inhibit the Aβ aggregation *via* binding with Aβ monomer and fibers. Thus, Ma-3-gal-Cl showed significant effects on protecting SH-SY5Y cells from Aβ/Cu^2+^/AA induced damage *via* antioxidation effect and inhibition effect to the Aβ aggregation.

## Introduction

Alzheimer's disease (AD) is a chronic neurodegenerative disease (1), which is most commonly associated with dementia in elderly people. Its clinical symptoms usually include memory loss, cognitive impairment, and behavioral dysfunction. The characteristic pathological changes in AD include cerebral atrophy, amyloid plaques, and neurofibrillary tangles in the brains of patients. The pathology of AD shows a significant correlation between amyloid-beta (Aβ) protein and the clinical severity of dementia (2). The amyloid cascade hypothesis indicates that the Aβ aggregates that self-assembled from misfolded Aβ can affect the structure and functions of neuronal cells and induce cell apoptosis, leading to synaptic dysfunction and neurodegeneration (3). Aβ peptides of 39–43 amino acids are a kind of hydrolysate of the transmembrane glycoprotein β amyloid precursor protein (APP). Among these Aβ peptides, Aβ (1–42) has the most prone to self-aggregate and generate neurotoxic aggregates (e.g., oligomers and fibers) (4). Meanwhile, the excess of copper ions (Cu^2+^) (~0.4 mmol/L), which were found in the senile plaque, were reported to induce oxidative stress in the AD brain, which is also an important cause of neurotoxicity (5, 6). Under a normal physiological environment, Aβ cannot self-aggregate due to the low concentration (5). However, in the pathological environment, Aβ concentration was increased that induced it to self-aggregate. At the same time, Cu^2+^ could bind to Aβ and form the Aβ-Cu^2+^ complex, which could react with ascorbic acid (AA) (500 μmol/L−10 mmol/L) in the brain to continuously generate excessive hydroxyl radicals (OH·) and reactive oxygen species (ROS) *via* Fenton and Harber–Weiss reactions (7). These ROS radicals will further increase the damage to neurons and aggravate the progression of AD (8).

Based on these mechanisms, many attempts have been made to discover new drugs that may intervene in these processes to relieve the symptoms of AD (9). There is no doubt that natural products provide abundant bioactive molecules for pharmaceutical chemists to screen and find suitable active ingredients and promote them to promising lead compounds further (10, 11). Anthocyanins, a category of polyhydroxy or polymethoxy derivatives of 2-phenylbenzopyran cations (12, 13), can be found in high abundance in natural plants. The main structure of anthocyanins contains two substituted benzene rings separated by an oxygen-containing heterocycle (14). According to the number and position of the substituents, 635 anthocyanins have been identified (14). The most common anthocyanins in natural plants are pelargonidin, cyanidin, peonidin, delphinidin, petunidin, and malvidin (13). In recent years, studies have shown that anthocyanins can show a variety of biological activities such as antioxidant, anti-inflammatory, and anti-apoptosis (15, 16). It has been pointed out that long-term dietary supplementation with anthocyanins can help reverse age-related cognitive deficits and neurological functions (17, 18). Furthermore, several studies have shown that anthocyanins can delay the occurrence and development of AD by inhibiting Aβ aggregation, neural cell apoptosis, and anti-inflammatory and antioxidation effects (17, 18). A berry plant rich in anthocyanins, *Vaccinium myrtillus* (bilberry), has attracted our interest. Therefore, an effective strategy was expected to find ingredients unreported that may fight against AD.

Cheminformatics is a new interdisciplinary technology with rapid growth in recent years. In the field of drug discovery, many cheminformatics methods such as physical and chemical property calculation, quantitative structure–activity relationship research (QSAR), similarity calculation, and scaffold hopping have been widely used in drug absorption, distribution, metabolism, excretion, and toxicity (ADMET) prediction, drug target prediction, and virtual screening of new molecular entities (19–21). In the field of food, cheminformatics methods are also gradually used in flavor prediction, food traceability, food fraud, and food detection (22–25). Especially, they have been more and more widely applied in the studies of discovering unreported compound entities and interpreting unconfirmed biological mechanisms from plants or foods (26, 27). Techniques based on cheminformatics can make full use of the information-processing ability of computers and quickly find answers from the vast chemical space. This will not only help researchers get the results quickly but also avoid knowledge-based one-sidedness at different levels of understanding.

In this study, we expect to design an efficient computational method based on cheminformatics to find the expected ingredients from bilberry. First, we need to collect all the chemical ingredients in bilberry and filter out the molecules that obviously did not meet the requirements by calculating the basic physical and chemical properties. Then, based on the advanced artificial intelligence models, a reasonable filter rule should be constructed, and the ingredients with essential drug-likeness properties in bilberry will be selected by scoring and sorting. Next, several advanced target prediction methods will be employed to predict the interaction between the ingredients and important targets related to AD. Through knowledge-based analysis and discrimination, the relevant ingredients that may show good possibility against AD are expected to be found. According to the molecular mechanisms of the pathogenesis and progress of AD, reasonable *in vitro* experiments will be designed to further verify the biological activity of the screened ingredients. Molecular docking will be subsequently used to simulate the docking of the validated ingredients to further elaborate the possible mechanism of action.

## Materials and methods

### Cheminformatics approaches

All the ingredients of bilberry were collected from the FooDB database (https://foodb.ca/, Version 1.0). The basic physical and chemical properties were calculated using ChemDes (28) and PyBioMed (29) toolkits, which were widely used in the drug design and enabled quick calculation of a variety of molecular descriptors. The properties used here included molecular weight (MW), number of hydrogen bond receptors (nHA), number of hydrogen bond donors (nHD), number of rotatable bonds (nRot), number of rings (nRing), and formal charge (fChar).

The ADMET prediction was conducted using ADMETlab (Version 1.0 and 2.0) (20, 30), which was the famous platform for early ADMET evaluation in drug development. The ADMET indicators used here included LogP, hERG, H-HT, Ames, ROA, Carcinogenicity, Respiratory, Non-Genotoxic_Carcinogenicity, and Genotoxic_Carcinogenicity_Mutagenicity. A detailed explanation of these indicators can be found at https://admetmesh.scbdd.com/explanation/index. By using KNIME software and Python programming, the rules for filtering ingredients are set as shown in Supplementary Figure S1. By sorting the calculated ADMET scores, the best ones will be listed at the top.

To construct a reasonable pipeline for the target prediction, we first reviewed publications and found that Aβ (1–42) (UniProt ID: P05067), AChE (P22303), and caspase-3 (UniProt ID: P42574) could be three important targets though they act as different roles in different Alzheimer's hypotheses. Then, we tried to search for target prediction tools driven by advanced artificial intelligence. SEA (31), SwissTargetPrediction (32), TargetNet (21), PPB2 (33), PharmMapper (34, 35), and SuperPred (36, 37) were employed to perform the target prediction for ingredients refined after the ADMET filtering, since this step was a rough prediction, and the scoring standards varied between different tools. At the same time, more possibilities were expected to be seen. We set a relatively loose threshold, that is, the first 20 hits were retained and then forwarded to analysis.

### Experimental materials

Ma-3-gal-Cl (cat. no. IP-0246) was purchased from Shanghai Tauto Biotechnology Co., Ltd. (Shanghai, China). Fetal bovine serum (FBS) (cat. no. 10270-106), Dulbecco's Modified Eagle Media: Nutrient Mixture F-12 (DMEM/F12) (1:1) (cat. no. C11330500BT), and 0.25% trypsin (cat. no. 25200-056) were purchased from Gibco (CA, USA). Aβ (1–42) (cat. no. 107761-42-2) for atomic force microscopy was purchased from American Peptide Company, Inc. (California, American). Active oxygen detection kit (cat. no. S0033S) and mitochondrial membrane potential detection kit 5,5′,6,6′-Tetrachloro-1,1′,3,3′-tetraethyl-imidacarbocyanine (JC-1) (cat. no. C2006) were obtained from Beyotime Biological Reagent Co., Ltd (Shanghai, China). Coumarin-3-carboxylic acid (CCA) (cat. no. C85603), ascorbic acid (AA) (cat. no. A800296), 3-(4,5-Dimethylthiazol-2-yl)-2,5-diphenyltetrazolium bromide (MTT) (cat. no. M2003), CuSO_4_ (cat. no. 209198), 30% H_2_O_2_ (cat. no. 10011208), and other reagents were purchased from Sigma-Aldrich Co. (St Louis, MO, USA). Cleaved caspase-3 (1: 1000, cat. no. ARG57512) and P-p38 (1: 500, cat. no. ARG51850) were obtained from Arigo (Hsinchu City, Taiwan, China), and p38 (1: 1000, cat. no. #9212) was obtained from Cell Signaling Technology (Danvers, MA, USA). The Ma-3-gal-Cl was first dissolved in DMSO (2 mmol/L). Then, it will be diluted to different concentrations by cell culture medium of DMEM/F12 (1:1) in cell-related experiments and ultrapure water in other experiments for further use. The Aβ (1–42) powder was first dissolved in 20 mmol/L NaOH to get the concentration of 500 μmol/L and then diluted to different concentrations by DMEM/F12 in cell-related experiments and PBS (pH = 7.4) in other experiments for further use. CCA powder was dissolved in PBS (20 mmol/L, pH = 9.0) and then adjust the pH to 7.4 with KH_2_PO_4_ solution, and the CCA concentration was calculated as 5 mmol/L. Thioflavin (ThT) was prepared into a concentration of 5 μmol/L by PBS (10 mmol/L, pH = 7.4).

### Cell viability assays

The undifferentiated human neuroblastoma cell line (SH-SY5Y) is a commonly used neuronal cell model for Alzheimer's disease (38–40). Undifferentiated SH-SY5Y was purchased from USA ATCC Company (Washington, DC, USA). SH-SY5Y cells were cultured in DMEM/F12 (1:1) basic medium containing 10% FBS and 1% penicillin and streptomycin mixture in a cell incubator under 5% CO_2_ at 37°C. The well-cultured cells were transferred to a sterile 96-well plate with approximately 1 × 10^4^ cells/well. Aβ (1–42), Aβ (1–42)/Cu/AA, or Ma-3-gal-Cl solutions were mixed and incubated with the SH-SY5Y cells for 24 h. The viability of the SH-SY5Y cells exposed to each solution was determined with an MTT assay.

### Detection of hydroxyl radical

Coumarin-3-carboxylic acid was used as a fluorescence probe for OH· determination (41) with a Hitachi F-4600 spectrofluorometer from Hitachi High-Tech Corporation (Tokyo, Japan). CCA fluorescence was recorded at 540 nm, with an excitation wavelength of 390 nm. The widths of the entrance and exit slits were both 5 nm. The OH·amounts in the solutions of Aβ (1–42) (10 μmol/L)/Cu^2+^ (5 μmol/L)/AA (1 mmol/L) mixture in the presence or absence of different concentrations of Ma-3-gal-Cl (0.5, 4, and 10 μmol/L) were detected with CCA probe. The fluorescence ratio was calculated as follows:


(1) $$\begin{array}{l} \text{Ratio }\left(\text{\%}\right)=100\times {\text{F}/\text{F}}_{0} \end{array}$$

Where F is the CCA fluorescence intensity in each solution, and F_0_ is the CCA fluorescence intensity of the Aβ (1–42) (10 μmol/L)/Cu^2+^ (5 μmol/L)/AA (1 mmol/L) mixture.

### Intracellular reactive oxygen species determination

DCFH-DA was used to detect the level of intracellular reactive oxygen species (ROS). The SH-SY5Y cells were treated with different solutions for 24 h and then treated according to the instruction of the DCFH-DA kit. The fluorescence intensity was recorded with the Hitachi F-4600 spectrofluorometer from Hitachi High-Tech Corporation (Tokyo, Japan) with the excitation and emission wavelengths at 485 nm and 525 nm. The fluorescence ratio was calculated as follows:


(2) $$\begin{array}{l} \text{Ratio }\left(\text{\%}\right)=100\times {\text{F}/\text{F}}_{0} \end{array}$$

Where *F* is the DCFH-DA fluorescence intensity of Aβ (1–42) (10 μmol/L)/Cu^2+^ (5 μmol/L)/AA (1 mmol/L)/Ma-3-gal-Cl (0.5, 4, 10 μmol/L) mixtures treated SH-SY5Y cells, and F_0_ is the DCFH-DA fluorescence intensity of the Aβ (1–42) (10 μmol/L)/Cu^2+^ (5 μmol/L)/AA (1 mmol/L) treated SH-SY5Y cells.

### Mitochondrial membrane potential determination

Fluorescent probe JC-1 was used for detecting the mitochondrial membrane potential (MMP) of SH-SY5Y cells. After the incubation treatment, the cells were treated with JC-1 dye following the instruction of the JC-1 kit. The fluorescence intensity was recorded with a DNM-9602 microplate reader from Plan New Technology Co., Ltd. (Beijing, China), and the MMP was calculated as the ratio of JC-1 monomer (FI 530)/JC-1 aggregate (FI 590) fluorescence intensity (42).

### Western blot analysis

Proteins were extracted from pretreated SH-SY5Y cells, and protein level was measured with a BCA protein quantification assay kit [Labgic Technology Co., Ltd. (Hefei, China)]. Proteins (8–12 μg/μL) were mixed with equal volume (8–12 μL) of sodium dodecyl sulfate (SDS) buffer (0.125 mol/L Tris-HCl, pH 6.8, 2% SDS, 0.5% 2-mercaptoethanol, 1% bromophenol blue, and 19% glycerol) and boiled for 5 min. Proteins were separated by SDS-polyacrylamide gel and transferred to nitrocellulose membranes. Then, the membranes were incubated with caspase-3 (cleaved), p38, and P-p38 antibodies overnight at 4°C, probed with horseradish peroxidase-conjugated secondary antibodies at room temperature, and imaged with Gel Imager 721-BR10883 from BIO-RAD Co., Ltd. (Hercules, CA, USA).

### ThT assays

Thioflavin was used for detecting the aggregation process of Aβ (1–42). The ThT fluorescence intensity of Aβ (1–42) (80 μmol/L) incubated with or without Ma-3-gal-Cl (10 μmol/L) at different times (0, 2, 4, 6, 12, 24, 48, 72, and 96 h) was detected with a Hitachi F-4600 spectrofluorometer (Hitachi, Japan) with the excitation and emission wavelengths of 450 and 490 nm, respectively. The widths of the entrance and exit slits were both 10 nm. The concentration of ThT was 5 μmol/L.

### Atomic force microscopy

Atomic force microscopy (AFM) can be used to observe the surface structure of Aβ (1–42) aggregates (43, 44). In this experiment, morphologies of Aβ (1–42) aggregates were characterized with a Nanoman vs. AFM (Bruker, Germany) with tapping mode. Aβ (1–42) was pretreated with hexafluoro-isopropyl alcohol (HFIP) and freeze-dried into powder. The freeze-dried powder was dissolved with 20 mmol/L NaOH and diluted into the experimental concentration with 10 mmol/L PBS buffer (pH 7.4) solution. Samples taken from incubated Aβ (1–42) solutions or Aβ (1–42)/Ma-3-gal-Cl mixtures were dropped on Ni^2+^-treated mica sheets, briefly washed with ultrapure water, and eventually dried under the gentle nitrogen stream.

### Molecular docking

The docking studies were performed using Molecular Operating Environment (MOE) software (version 2019). Before the docking study, we first prepared the Ma-3-gal-Cl by searching the conformation using the default parameters. Then, for proteins, water molecules, ions, and non-standard amino acid residues were detached from the proteins and the hydrogen atoms were added under an AMBERT10 force field. After the automated correction of protein structure using the “Structure Preparation” module, the binding sites were detected. For caspase-3, crystal structure 2J33 (PDB ID: 2J33) (45) was selected as the unactivated model, and the binding sites were selected according to the original ligand. For the crystal structure of MKK6 (PDB ID: 3VN9) (46), the prior site could be ATP binding site. For the Aβ crystal structure of monomer (PDB ID: 1IYT) (47) and fibril (PDB ID: 2BEG) (48), we scanned the whole surface to find better sites. The prepared Ma-3-gal-Cl was then flexibly docked into the receptor using the “Triangle Matcher” placement method and “London dG” scoring with other default parameters. Finally, ten docking poses were obtained, and the one with the best score was chosen for analysis.

### Statistical analysis

The results were calculated using the SPSS l7.0 statistical analysis, and Origin was used for drawing. Each experiment and each group of data were repeated three times. The results were expressed by mean ± standard deviation (mean ± SD), and the single-sample *t*-test was used to compare the group differences. *P* < 0.05 indicated a statistical difference.

## Results

### Basic screening of drug-likeness ingredients

From the FooDB database, 4,143 components of bilberry were collected. From these components, glycerol and fatty acids such as triglyceride (TG), diacylglycerol (DG), and phosphatidyl ethanolamine (PE) were preliminarily deleted, and 182 ingredients were obtained. After removing 38 entries such as water, inorganic substances, and triglycerides, 144 ingredients were obtained. Next, we retained the ingredients with a molecular weight of <100 because compounds with very small molecular weights always behave without specificity. After this, 132 ingredients were retained for the next analysis.

The 132 ingredients were then fed into the pipeline for ADMET and drug-likeness screening. As the process described in the Python code (Supplementary Figure S1), this step adopted the strategy of a cumulative score. Each ingredient got an inherent score according to whether the properties meet the requirements, and finally each ingredient got a total score. After ranking the total scores, we get the top 10 ingredients. The structures of these 10 ingredients are listed in Supplementary Table S1. We found that delphinidin (49), cyanidin (50), petunidin (51), quercetin (52), gallocatechin (53), epigallocatechin (54), and myricetin (55) all have been reported to relate with their function against AD, while ingredients based on Malvidin scaffold seem no specific reports. This led to our way of thinking: can we make further study of malvidin-3-O-galactoside (Malvidin scaffold) to see whether it is a neuroprotective ingredient that will help to complete a full profile of the bilberry against AD?

### Prediction and selection of ingredients against AD

**Figure 1:**
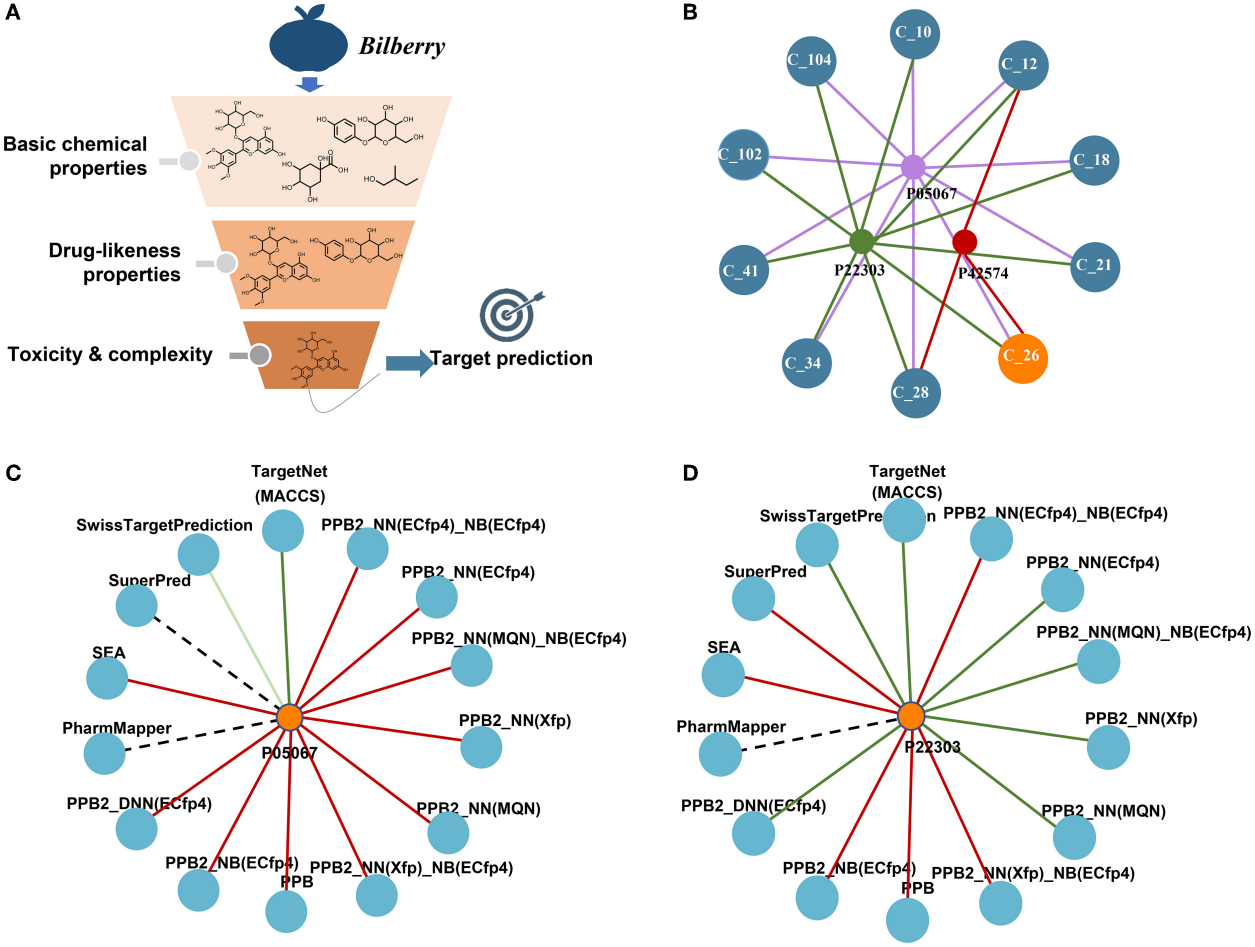
Screening of neuroprotective ingredients by cheminformatics approaches. **(A)** The schema of the pipeline. **(B)** The predicted targets and their interactions with the top 10 compounds. C_26 represents Ma-3-gal-Cl. **(C)** The predicted interactions between Ma-3-gal-Cl and Aβ (1–42) by different tools. The red line represents no hit, the dark green represents a hit, and the light green line represents a hit out of the top 20. The dotted line means the tool does not cover this target. **(D)** The predicted interactions between Ma-3-gal-Cl and AChE by different tools.

The target prediction results are shown in Figure 1. The simple network displaying the interaction between the top 10 compounds including Ma-3-gal-Cl and the three targets was constructed as shown in Figure 1B. The top 10 compounds all process the possibility that could be interacted with the targets, which was somehow confirmed by the publications mentioned above. For Ma-3-gal-Cl (number C_26), Aβ (1–42) (P05067), Caspase-3 (P42574), and AChE (P22303) were predicted as its targets. To enhance the reliability, we compared the prediction results of different tools. From Figures 1C,D, we observe that TargetNet and SwissTargetPrediction voted the Ma-3-gal-Cl to interact with Aβ (1–42), and TargetNet, SwissTargetPrediction, and PPB2 voted the Ma-3-gal-Cl to interact with AChE. Although the unified prediction criteria and degree of different tools cannot be implemented, the voting results will provide evidence of interaction from multiple perspectives (e.g., similarity, pharmacophore, and chemical genomics). This increased our confidence in exploring its mechanisms against AD related to these two targets.

In AD patients' brains, Aβ was complexed with excess metal (such as copper, iron, and aluminum) ions in senile plaques. It was reported that the Aβ-metal complex, especially the Aβ-Cu^2+^ complex, can facilitate the generation of H_2_O_2_ by reacting with cellular species such as AA and O_2_ (56, 57). In the cellular milieu, any rogue Cu^2+^ that is not readily complexed by Aβ will also react with H_2_O_2_ to produce hydroxyl radicals *via* the Harber–Weiss reaction and induce oxidative stress to neurons. Meanwhile, the toxic Aβ aggregates are also one of the culprits for neuron apoptosis. According to the cheminformatics results, Ma-3-gal-Cl could interact with Aβ, which suggested that Ma-3-gal-Cl may influence Aβ-Cu^2+^ induced oxidative stress to cells and the formation of the toxic Aβ aggregates. Thus, *in vitro* experiments were performed.

### The inhibitory effect of Ma-3-gal-Cl on oxidative damage induced SH-SY5Y cell apoptosis

**Figure 2:**
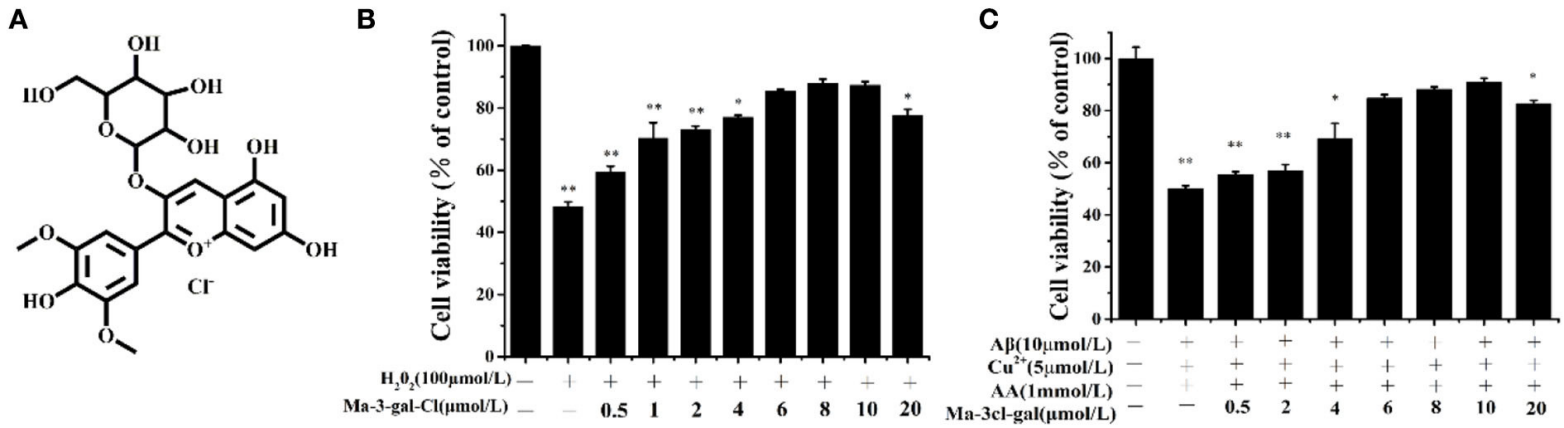
Molecular structure of Ma-3-gal-Cl **(A)**, and cell viability of SH-SY5Y cells treated with different solutions **(B,C)**. ^*^p < 0.05 and ^**^p < 0.01 compared to control values.

H_2_O_2_ is an important reactive oxygen species (ROS), and it is also a precursor for other ROS (e.g., HO). In order to explore the protective effect of Ma-3-gal-Cl on oxidative damage of H_2_O_2_ to SH-SY5Y cells, SH-SY5Y cells were incubated with different concentrations of Ma-3-gal-Cl and H_2_O_2_ for 24 h, and the cell viability of SH-SY5Y cells was detected by MTT assay. The results showed that Ma-3-gal-Cl could effectively improve the cell viability of SH-SY5Y cells treated with H_2_O_2._ When the concentration of Ma-3-gal-Cl increased from 0 to 10 μmol/L, the cell viability was increased from 48.2 to 87.3% (*P* < 0.01) (Figure 2B). Additionally, when the concentration of Ma-3-gal-Cl increased to 20 μmol/L, the cell viability was decreased to 77.6%. These results indicated that Ma-3-gal-Cl with a lower concentration (below 10 μmol/L) could effectively inhibit the H_2_O_2−_induced cell apoptosis. But at the high concentration of Ma-3-gal-Cl (20 μmol/L), due to the cell toxicity of Ma-3-gal-Cl, lower cell viability was observed (Supplementary Figure S2).

In the senile plaques of patients with AD, large amounts of redox-active metal ions such as Cu^2+^ and Fe^2+^ have been found to coexist with the aggregates of amyloid-beta (Aβ) peptides (58). These metal ions can strongly bind Aβ peptides (58, 59), and the resultant complexes can facilitate the generation of H_2_O_2_ by reacting with cellular species such as AA and O_2_ (59). Furthermore, redox metal ions (e.g., Cu^2+^ and Fe^2+^) can generate hydroxyl radicals (OH**·**) by reacting with H_2_O_2_ through the Harber–Weiss and Fenton reactions (8, 60). Thus, in this study, the Aβ (1–42)/Cu^2+^/AA system was used as the OH**·** production model to simulate the OH**·** producing process in senile plaques of patients with AD.

To investigate the inhibition effect of Ma-3-gal-Cl to Aβ (1–42)/Cu^2+^/AA induced cell apoptosis, the cell viability of SH-SY5Y cells incubated with different concentrations of Ma-3-gal-Cl/Aβ (1–42)/Cu^2+^/AA mixture was detected. As shown in Supplementary Figure S3, the low concentrations of Cu^2+^ (5 μmol/L) or AA (1 mmol/L) alone showed no cell toxicity to SH-SY5Y cells. Aβ (1–42) (10 μmol/L) reduced the cell viability to 92.3% (Supplementary Figure S3). However, when the same concentration of Aβ (1–42), Cu^2+^, and AA was mixed, the cell viability was decreased to 55.0%. This may be due to the oxidative damage from OH· produced by Aβ (1–42)/Cu^2+^/AA system, and the cell toxicity from Aβ (1–42) toxic aggregates. As shown in Figure 2C, when the concentration of Ma-3-gal-Cl increased from 0 to 10 μmol/L, the cell viability was increased from 55.0% to 90.9%. When the concentration of Ma-3-gal-Cl increased to 20 μmol/L, the cell viability was decreased to 82.73%. These results indicated that when the concentration of Ma-3-gal-Cl is below 10 μmol/L, it could inhibit the Aβ (1–42)/Cu^2+^/AA system so as to induce cell apoptosis.

### Scavenging effect of Ma-3-gal-Cl on OH· produced by Aβ (1–42)/Cu^2+^/AA system

**Figure 3:**
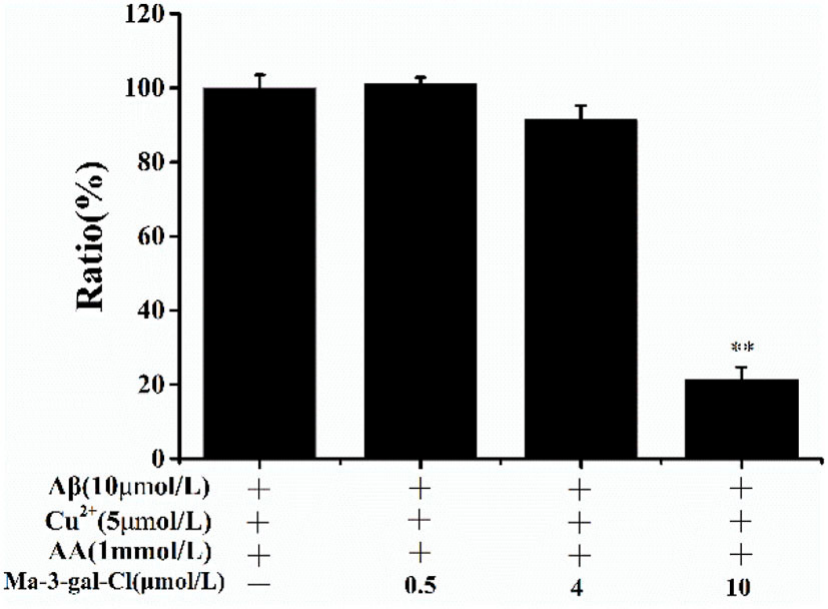
CCA fluorescence ratio of Aβ (1–42)/Cu^2+^/AA solutions in the absence or presence of different concentrations of Ma-3-gal-Cl. ^**^p < 0.01 compared to values obtained in Aβ (1–42)/Cu^2+^/AA solution.

To investigate the mechanism of the protective effect of Ma-3-gal-Cl to Aβ (1–42)/Cu^2+^/AA mixture treated cells, the scavenging effect of Ma-3-gal-Cl on OH· produced by Aβ (1–42)/Cu^2+^/AA mixture was detected with the CCA method (61). With the increase in Ma-3-gal-Cl concentration (0, 0.5, 4, and 10 μmol/L), the amount of the OH· decreased from 100 to 20% (Figure 3). These results indicate that Ma-3-gal-Cl could inhibit the production of OH· by Aβ (1–42)/Cu^2+^/AA mixture, and the inhibition effect is concentration-dependent.

### Effects of Ma-3-gal-Cl on intracellular ROS production and mitochondrial membrane potential in Aβ (1–42)/Cu^2+^/AA-treated SH-SY5Y cells

**Figure 4:**
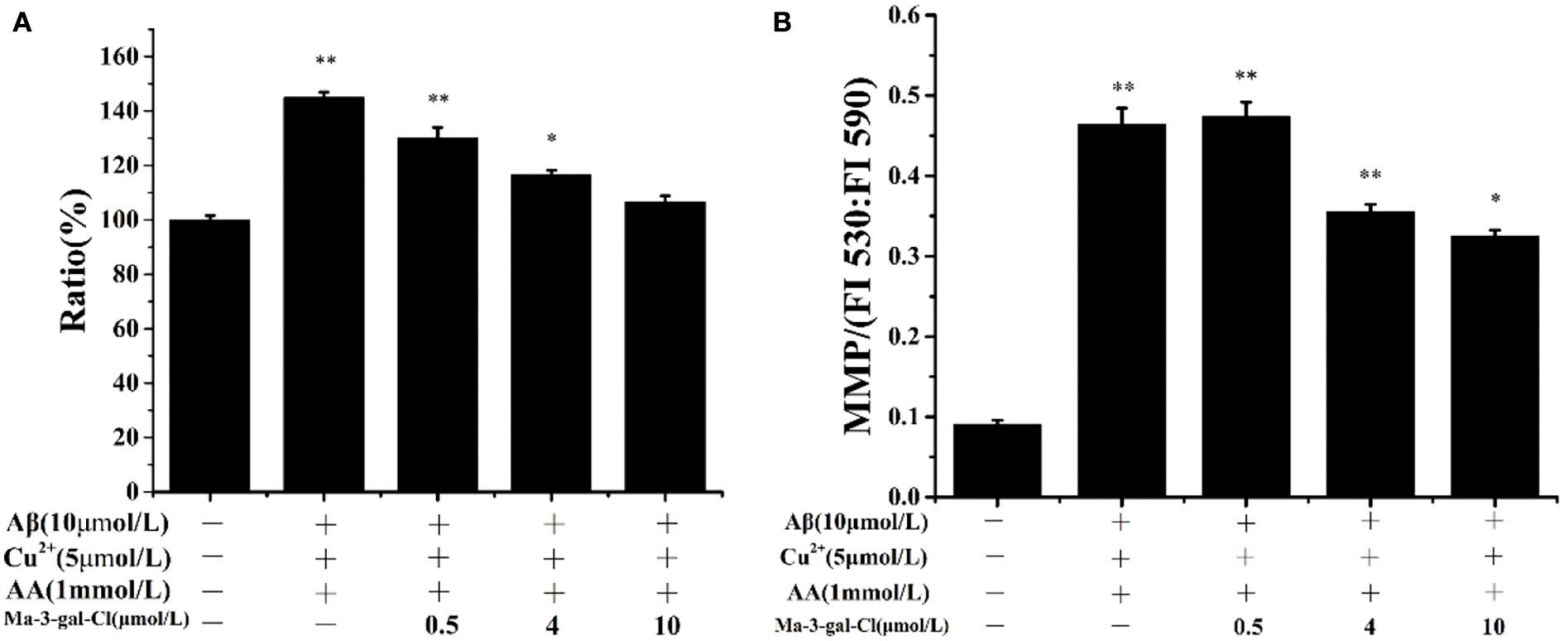
Intracellular ROS ratio **(A)** and MMP **(B)** of SH-SY5Y cells which were treated with Aβ (1–42)/Cu^2+^/AA mixture in the absence or presence of different concentrations of Ma-3-gal-Cl. ^*^P < 0.05 and ^**^p < 0.01 compared to control values.

Intracellular ROS level is an important reflection of oxidative damage, and it can be detected with DCFH-DA (62). As shown in Figure 4A, the amount of intracellular ROS in SH-SY5Y cells incubated by Aβ (1–42)/Cu^2+^/AA increased to 145%. With the increase in Ma-3-gal concentration (0–10 μmol/L), the amount of intracellular ROS was reduced (from 145.0 to 106.7%, compared with the control group). These results indicated that Ma-3-gal-Cl can reduce the intracellular ROS production of SH-SY5Y cells induced by Aβ (1–42)/Cu^2+^/AA mixture.

Oxidative stress can lead to the decline of the MMP of cells and result in cell apoptosis (63, 64). To study the protective effect of Ma-3-gal-Cl on the Aβ (1–42)/Cu^2+^/AA treated SH-SY5Y cells, the MMP was determined with JC-1 staining (42). As shown in Figure 4B, the MMP could be increased dramatically by Aβ (1–42)/Cu^2+^/AA from 0.092 to 0.464. With the addition of Ma-3-gal-Cl, the MMP values were decreased. When the concentration of Ma-3-gal-Cl was at 4 μmol/L, the MMP value decreased to 0.356 and further increased the Ma-3-gal-Cl concentration to 10 μmol/L, and the MMP value only decreased to 0.326. These results indicated that Ma-3-gal-Cl could reduce the mitochondrial damage caused by oxidative stress from Aβ (1–42)/Cu^2+^/AA.

### Effects of Ma-3-gal-Cl on expression of caspase-3 and p38 in Aβ (1–42)/Cu^2+^/AA-treated SH-SY5Y cells

**Figure 5:**
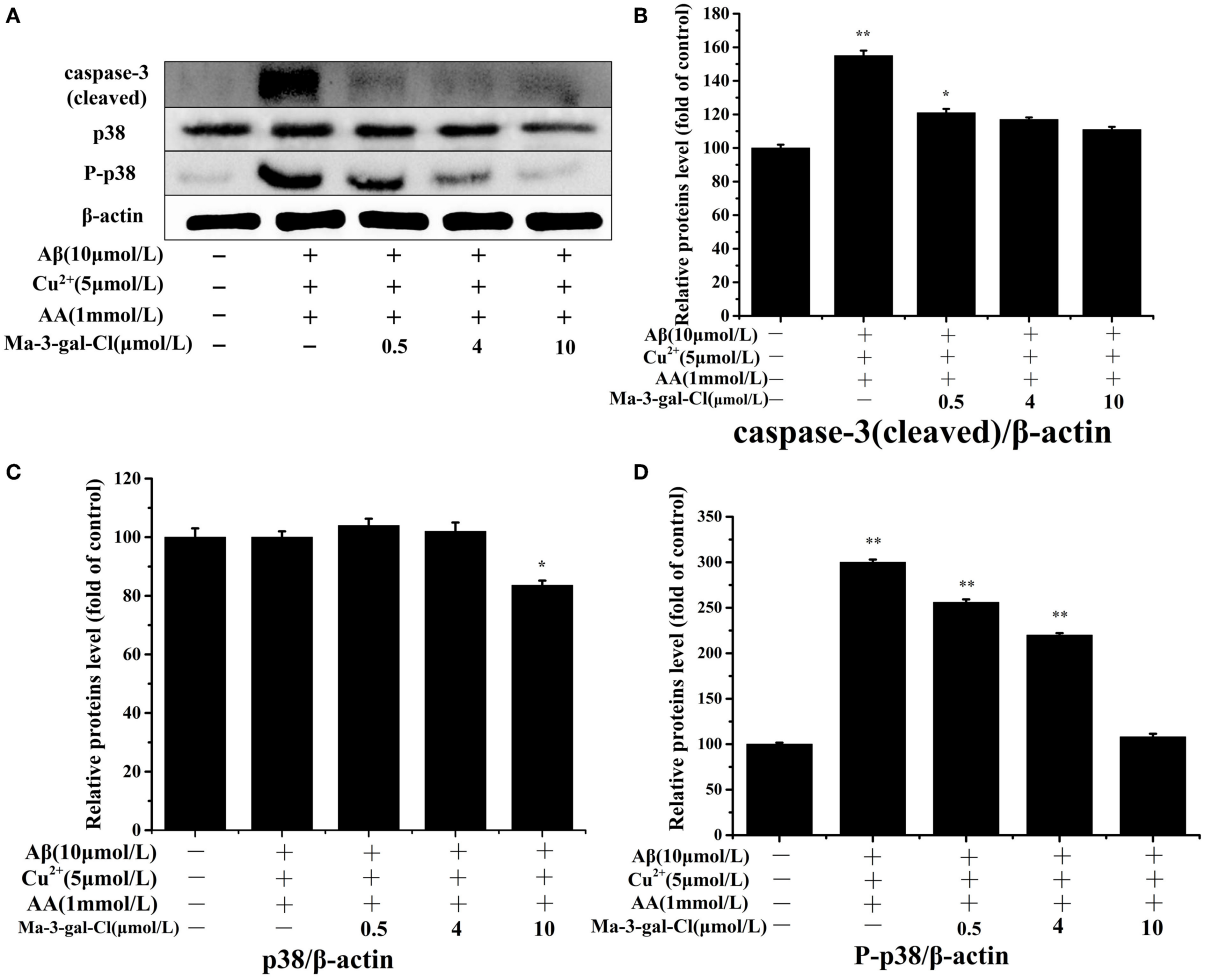
Western blot results **(A)** and effect of Ma-3-gal-Cl on the expression of caspase-3 (cleaved) **(B)**, p38 **(C)**, and P-p38 **(D)** in pretreated SH-SY5Y cells. *P < 0.05 and **p < 0.01 compared to control values.

The caspase-3 and p38 are important proteins in the apoptosis pathway, and their activations can trigger a series of apoptosis reactions and lead to cell apoptosis. To investigate the molecular mechanism of the inhibition effect of Ma-3-gal-Cl to SH-SY5Y apoptosis induced by Aβ (1–42)/Cu^2+^/AA, different concentrations (0.5, 4, and 10 μmol/L) of Ma-3-gal-Cl were selected, and the expression level of target protein was detected by Western blot (WB) assay. As shown in Figures 5A,B, the expression level of the caspase-3 (cleaved) increased in the Aβ (1–42)/Cu^2+^/AA treated SH-SY5Y cells (from 100 to 155%), and the one for Ma-3-gal-Cl/Aβ (1–42)/Cu^2+^/AA mixture treated cells was decreased (from 155 to 111%). As shown in Figures 5A,C, compared with the control group, the Aβ (1–42)/Cu^2+^/AA mixture and low concentration (below 4 μmol/L) of Ma-3-gal-Cl almost have no influence on the expression of p38 in cells (Figure 5C). However, Aβ (1–42)/Cu^2+^/AA incubation could increase the expression of P-p38 in SH-SY5Y cells. Additionally, Ma-3-gal-Cl (0–10 μmol/L) could dramatically decrease the P-p38 expression (from 300 to 108%) (Figure 5D). These results indicate that Ma-3-gal-Cl could not influence the expression of p-38 in Aβ (1–42)/Cu^2+^/AA treated SH-SY5Y cells, but it could inhibit the expression of cleaved caspase-3 and P-p38 in Aβ (1–42)/Cu^2+^/AA treated SH-SY5Y cells.

### The inhibition effect of Ma-3-gal-Cl on Aβ (1–42) aggregation

**Figure 6:**
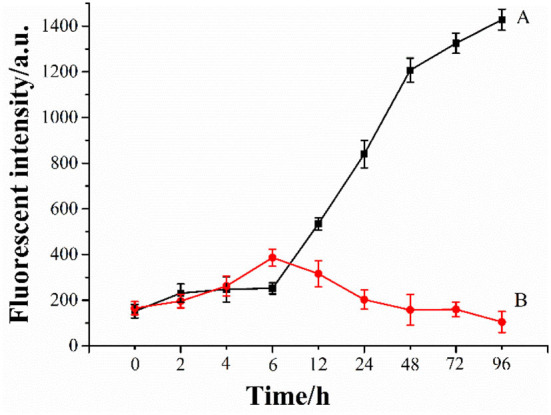
ThT fluorescence intensity of Aβ (1–42) (80 μmol/L) (A) and Aβ (1–42) (80 μmol/L)/Ma-3-gal-Cl (10 μmol/L) (B) mixture at different time.

The aggregates (oligomers, protofibrils, and fibrils) of Aβ were considered toxic species to neuron cells (65, 66). To investigate the inhibition effect of Ma-3-gal-Cl on Aβ aggregation, a ThT assay was employed. As shown in Figure 6, line A, in the first 6 h, the fluorescent intensity of the Aβ (1–42) solution increased slowly. When the incubation time is at the range of 6–48 h, the fluorescent intensity increased sharply, and the one for the longer incubation time (48–96 h) increased gradually again. These results are consistent with other reports (67–69). The lag phase (ca. 6 h) is indicative of the nucleation phase for Aβ (1–42) aggregation, whereas the fluorescent intensity plateau (48–96 h) corresponds to the formation of well-ordered, β-sheet-rich Aβ (1–42) fibrils. When the Ma-3-gal-Cl was incubated with Aβ (1–42), the fluorescent intensity of ThT increased gradually at the first 6 h and decreased at later times (Figure 6, line B). These results indicated that Ma-3-gal-Cl could inhibit the aggregation of Aβ (1–42) at the long incubation time, but could not completely inhibit Aβ (1–42) aggregation in the early hours of the incubation.

**Figure 7:**
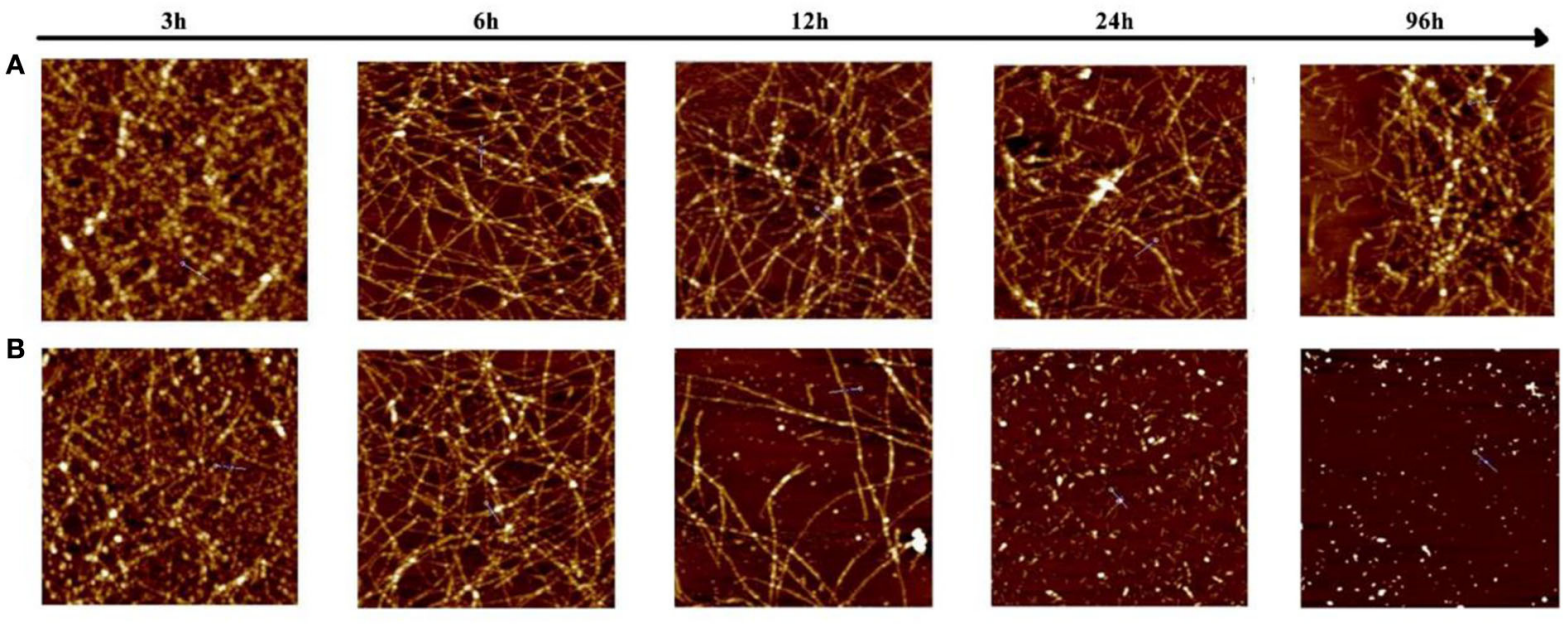
Atomic force microscopy (AFM) images of samples taken from an 80 μmol/L Aβ (1–42) solution **(A)** and Aβ (1–42) (80 μmol/L)/Ma-3-gal-Cl (10 μmol/L) mixture **(B)** at different incubation times. The scale is 2 × 2 μm.

To investigate the morphology of products from the Aβ (1–42) and Ma-3-gal-Cl/Aβ (1–42) mixture, the atomic force microscope (AFM) was employed. In the Aβ (1–42) solutions, globular aggregates and short protofibrils were observed in the 3-h-incubation sample (cf. images juxtaposed in row A of Figure 7), which is consistent with the lag phase in Figure 6. After 6 h, more protofibrils were produced, and after 24 h, mature Aβ (1–42) fibrils were dominated. In the presence of Ma-3-gal-Cl, at the first 6 h, the morphology of the Aβ (1–42) aggregates (Figure 7, row B) was similar to those in Aβ (1–42) alone solution (Figure 7, row A), but at 12 h, the density of Aβ (1–42) fibrils was decreased and after 24 h, the mature fibrils could not be observed, and instead with short, small aggregates. At 96 h, the amount of the small aggregates dramatically decreased. These results were consistent with ThT fluorescence results (Figure 6). This observation suggested that Ma-3-gal-Cl could bind with the Aβ (1–42) monomer and large oligomers to prevent Aβ (1–42) oligomers and protofibrils from further growing into mature fibrils.

### The molecular docking results and analysis

In order to explore the specific structural basis and molecular mechanism of antioxidant and anti-aggregation effects of Ma-3-gal-Cl to Aβ (1–42), the molecular docking method was employed for further study. In the *in vitro* tests, the expressions of activated caspase-3 and P-p38 of Ma-3-gal-Cl/Aβ (1–42)/Cu^2+^/AA mixture treated cells were decreased (Figure 5). The Aβ (1–42) aggregates were also inhibited by Ma-3-gal-Cl (Figures 6, 7). Thus, the unactivated caspase-3 (crystal structure of 2J33), mitogen-activated protein kinase kinases-6 (MKK6) (PDB ID: 3VN9) related with the active process of p-38, Aβ monomer (PDB ID: 1IYT), and fibril (PDB ID: 2BEG) were selected as receptors for the molecular docking experiments.

**Figure 8:**
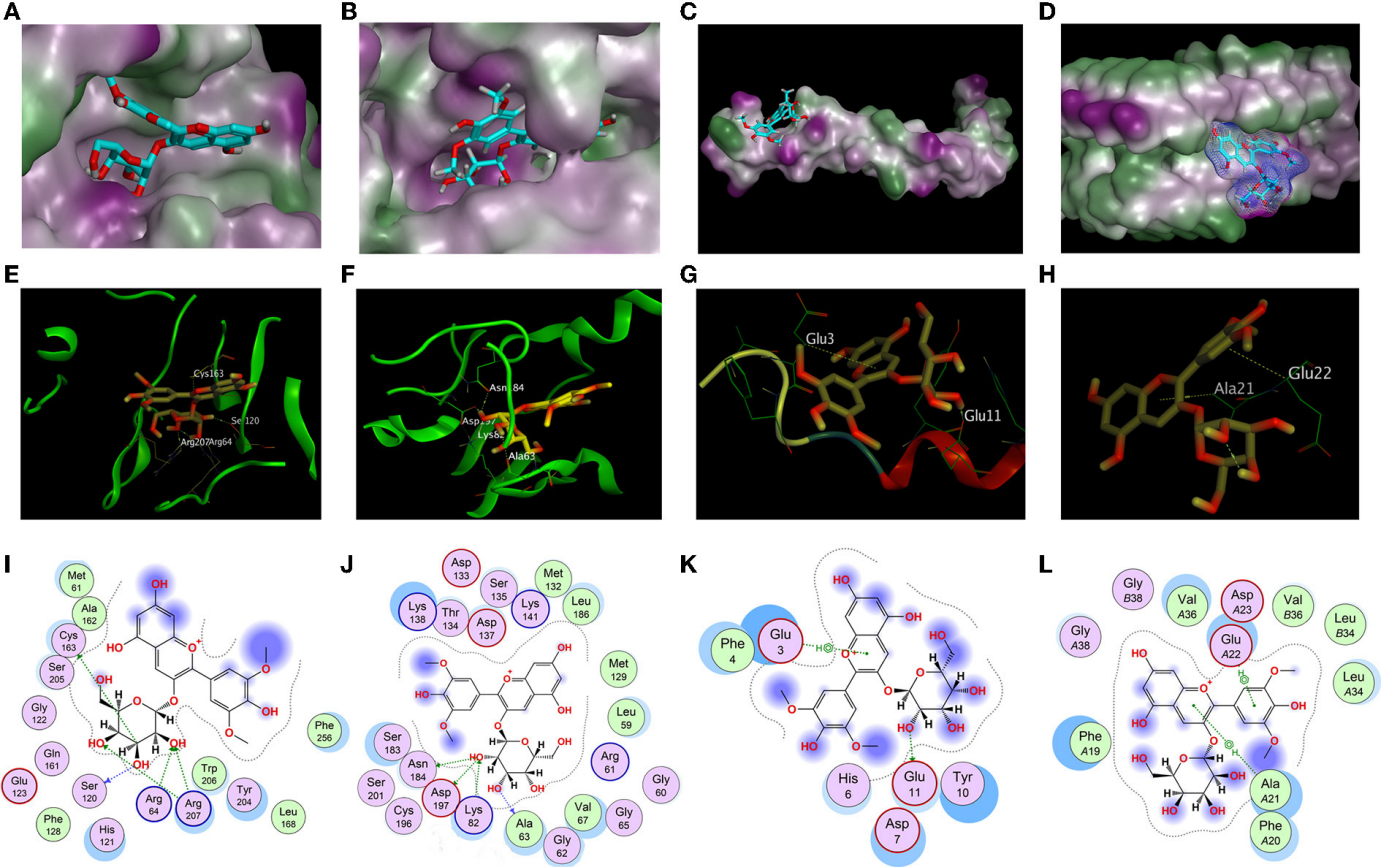
Docking results of Ma-3-gal-Cl. **(A,E,I)** The best binding pose, side view, and interaction graph of Ma-3-gal-Cl docked into the crystal structure of caspase-3 (PDB ID: 2J33). **(B,F,J)** The best binding pose, site view, and interaction graph of Ma-3-gal-Cl docked into the crystal structure of MKK6 (PDB ID: 3VN9). **(C,G,K)** The best binding pose, site view, and interaction graph of Ma-3-gal-Cl docked into the crystal structure of Aβ (1–42) monomer (PDB ID: 1IYT). **(D,H,L)** The best binding pose, side view, and interaction graph of Ma-3-gal-Cl docked into the crystal structure of Aβ (1–42) fibril (PDB ID: 2BEG).

The structure-based docking of unactivated caspase-3, MKK6, Aβ (1–42) monomer, and fibril with Ma-3-gal-Cl gave the best score of −7.12, −7.68, −5.51, and −6.52 kCal/mol, respectively. Their interactions were visualized in 2D and 3D diagrams, and the hydrogen bond interactions between Ma-3-gal-Cl and the active site residues were observed (Figure 8). When processing the docking caspase-3 with Ma-3-gal-Cl, we first made a self-docking using the ligand in the crystal structure. The RMSD between the best pose and the original ligand is 1.41 Å, which indicated a set of proper docking parameters for this system. By using this set of parameters, we found that the residues, namely Cys163 (bond length: 3.60 Å), Ser120 (bond length: 2.92 Å), Arg207 (bond length: 2.77 Å), Arg64 (bond length: 2.96 Å), and Arg207 (bond length: 3.00 Å), were observed to form five hydrogen bonds with Ma-3-gal-Cl of the caspase-3 receptor (Figures 8A,E,I). For MKK6, Ma-3-gal-Cl was docked to the ATP binding site which indicated an ATP-site-directed inhibitor (70, 71). The residues, namely Ala63 (bond length: 3.20 Å), Asn184 (bond length: 2.95 Å), Asp197 (bond length: 3.03 Å), and Lys82 (bond length: 2.88 Å), were observed to form four hydrogen bonds (Figures 8B,F,J). Ma-3-gal-Cl could stay at the site with a good pose. For Aβ (1–42) monomer, there were no obvious pockets with good geometry and hydrophobicity because of no complex secondary structures. However, we found that most of the retained 10 poses tended to interact with N-terminus residues (3–17), while the importance of the N-terminal residues has been indicated in oligomerization and neurotoxicity (72, 73). In addition, these regions are the essential components of the binding site for glycosaminoglycans, which affects the change in Aβ (1–42) secondary structure from a soluble α-helix conformation to a stable β-sheet one. In the best pose, a hydrogen bond (Glu11) and a pi–H interaction (Glu3) (Figures 8C,G,K) were observed. For Aβ (1–42) fibril, the NMR structure gives important information about the identification of interaction regions, which might be targeted by inhibitor compounds. It suggested specific structural properties to interact and destabilize Aβ (1–42) self-assembly, including (a) the hydrophilic region caused by electrostatic interaction between Asp23-Lys28, (b) the Glu22 ladder formed by the Glu22 residue side chains of adjacent β-sheets, (c) the central cleft in the interior of the U-shaped turn, and (d) the hydrophobic regions caused by Leu17-Ala21 and Ala30-Val36 residues, respectively. The best pose in our study occupied the central cleft in the interior of the U-shaped turn and formed two pi–H interactions (Ala21 and Glu22), which was in line with the proposed features (Figures 8D,H,L). In addition, three poses from the 10 retained ones tended to form pi–H interactions with Glu22, Phe19, and Phe20. This can be corroborated from the side by a recent study, which indicated the π-π interactions of inhibitor agents with Phe19 and Phe20 residues can stabilize the fibrils by a reduction in the total energy of the conformation (74).

The docking results indicate that Ma-3-gal-Cl could interact with unactivated caspase-3, MKK6, Aβ (1–42) monomer, and fibril. Although there were differences in scores and interactions, there was a relatively high degree of agreement with the evidence provided by published studies. Furthermore, the calculated results accord with the experiments, which reflected our reasonable hypothesis that Ma-3-gal-Cl could reduce neuronal apoptosis by combining antioxidation and anti-aggregation of Aβ (1–42).

## Discussion

A key step in exploring the active ingredients of bilberry by using cheminformatics methods is how to narrow the scope by using the basic physical and chemical properties and ADMET properties, so as to obtain the top-ranked compounds for further study. In fact, this rule should be customized according to different projects and purposes, and of no fixed pattern. In this work: (1) the molecular size was restricted because too small molecules often have no specificity; (2) the synthesizability was considered. If the synthesis is too easy, the molecule is often too simple. If the synthesis is too difficult, it is often not enough economy and feasibility. (3) Restricting indicators related to toxicity is to meet the potential of a drug. (4) Defined the commonly seen structural descriptors, including hydrogen bonds, rings and bonds, and logP, so that they will conform to the drug-likeness. (5) In general, specific and strict ADMET screening rules are usually used in drug optimization or specific aim. In the preliminary activity explore of food ingredients, it is appropriate to limit only the basic physical and chemical properties and toxicity.

According to these cheminformatics approaches, Ma-3-gal-Cl was screened from bilberry. Meanwhile, cheminformatics results also suggested Ma-3-gal-Cl could interact with Aβ. Owing to that the oxidative stress induced by the Aβ-Cu^2+^ complex and the toxic Aβ aggregates could induce neuron apoptosis and further induce AD, the effects of Ma-3-gal-Cl on these two pathways related to Aβ were investigated with *in vitro* studies.

Before the experimental investigation, we also evaluated the ability to penetrate across the blood–brain barrier (BBB) of Ma-3-gal-Cl. Ionic drugs usually need the assistance of transporters to pass through the blood–brain barrier (75–77). Referring to the method of Liu (78), we investigated the possibility of Ma-3-gal-Cl transit through the neural high-affinity chord transporter 1 (ChT1). They used the homology model based on the template of (vSGLT) because of the absence of crystal structure of ChT1 at that time. Here, based on the latest crystal structure predicted by AlphaFold, we used MOE software to evaluate the binding possibility between Ma-3-gal-Cl and BBB-ChT1 using rigid docking and semi-flexible docking (induced fit), respectively. The results showed that the binding energies of rigid and semi-flexible docking with the best scoring conformation were −7.8651 and −8.3885 kCal/mol, respectively, which indicated a potential penetrate across the BBB compared with the commonly used threshold (77, 78).

According to the *in vitro* experimental results, we believe that Ma-3-gal-Cl has the inhibition effect on Aβ/Cu^2+^/AA induced SH-SY5Y cell apoptosis, which is mainly caused by antioxidation and anti-Aβ aggregation process. In the brain of patients with AD, aggregated Aβ and the excess amount of Cu^2+^ (~0.4 mmol/L) were found in the senile plaque (79, 80). The toxic Aβ aggregates and oxidative stress produced by the Aβ/Cu^2+^ mixture/complex have been suggested to play an important role in AD pathogenesis (59).

As reported that the Cu^2+^ can strongly bind with Aβ peptides and the resultant complexes can facilitate the generation of H_2_O_2_ by reacting with cellular species such as AA and O_2_ (56):


(3) $$\begin{array}{l} \text{Ascorbic Acid}+\text{2A}\beta -\text{ ​}{\text{Cu}}^{\text{2+}}\\ +{\text{H}}_{\text{2}}\text{O}\to \text{Dehydroascorbic acid }{\text{+2H}}^{\text{+}}\text{+2A}\beta -{\text{Cu}}^{\text{+}} \end{array}$$

(4) $$\begin{array}{l} 2\text{A}\beta -{\text{Cu}}^{+}+{\text{O}}_{2}+2{\text{H}}^{+}\to 2\text{A}\beta -{\text{Cu}}^{2+}+{\text{H}}_{2}{\text{O}}_{2} \end{array}$$

In the cellular milieu, any rogue Cu^2+^ that is not readily complexed by Aβ will also react with H_2_O_2_ to produce hydroxyl radicals *via* Harber–Weiss reaction as (81):


(5) $$\begin{array}{l} {\text{H}}_{2}{\text{O}}_{2}+{\text{Cu}}^{2+}\to {\text{Cu}}^{+}\cdot {\text{O}}_{2}\text{H}\cdot +{\text{H}}^{+} \end{array}$$

(6) $$\begin{array}{l} {\text{Cu}}^{+}\cdot {\text{O}}_{2}\text{H}\cdot +{\text{H}}_{2}{\text{O}}_{2}\to {\text{Cu}}^{+}+{\text{O}}_{2}+{\text{OH}}^{\cdot }+{\text{H}}_{2}\text{O} \end{array}$$

(7) $$\begin{array}{l} {\text{H}}^{+}+{\text{Cu}}^{+}+{\text{H}}_{2}{\text{O}}_{2}\to {\text{Cu}}^{2+}+{\text{OH}}^{\cdot }+{\text{H}}_{2}\text{O} \end{array}$$

As shown in our work, Aβ (1–42)/Cu^2+^/AA system produced the hydroxyl radicals (Figure 3). The Ma-3-gal-Cl could inhibit the hydroxyl radical production and protect the SH-SY5Y cells from the oxidation damage induced by hydroxyl radicals (Figure 3). The amount of intracellular ROS and mitochondrial membrane potential in SH-SY5Y cells incubated with Aβ (1–42)/Cu^2+^/AA system were reduced by the Ma-3-gal-Cl treatment (Figure 4). The cell apoptosis of SH-SY5Y induced by the H_2_O_2_ or Aβ (1–42)/Cu^2+^/AA system was also inhibited by the Ma-3-gal-Cl treatment (Figure 2). Similar results were obtained by Kim et al. (82). In their study, three anthocyanin compounds extracted from black soybean (cyanidin-3-*O*-glucoside, delphinidin-3-*O*-glucoside, and petunidin-3-*O*-glucoside) were found could reduce the cytotoxicity of H_2_O_2_ to SK-N-SH cells and decrease intracellular ROS level. The antioxidant property of the anthocyanin compounds may be due to the phenolic hydroxyl groups (83).

The caspase family plays a key role in cellular apoptosis induced by oxidative damage and Aβ aggregates injury (84, 85). Caspase-3 is one of the most important downstream caspases in the apoptotic pathway, but the pro-caspase-3 has no activity in inducing cell apoptosis, while the cleaved caspase-3 has the activity. In our study, Ma-3-gal-Cl could bind with pro-caspase-3 (unactivated) (Figure 8) and inhibit the expression of cleaved caspase-3 (activated) (Figure 5).

Mitogen-activated protein kinase (MAPK) plays an important role in cell apoptosis, and the p38MAPK signaling pathway is responsible for transducing inflammatory signals and initiating apoptosis induced by oxidative damage and Aβ aggregates injury (86, 87). In the Alzheimer's disease (AD) brain, increased levels of phosphorylated (active) p38 were detected relative to age-matched normal brain (88), and the MKK6 is the main kinase for the phosphorylation process of p-38. In our study, Ma-3-gal-Cl was docked to the ATP binding site in MKK6 (Figure 8), which could inhibit the activity of MKK6 and reduced the amount of the phosphorylated (active) p38 (Figure 5).

In the senile plaque of the AD brain, aggregated Aβ is the main constituent (89). The Aβ aggregates, self-assembled from misfolded Aβ peptides, affect the structure and functions of neural cells and stimulate cell apoptosis, leading to synaptic dysfunction and neurodegeneration (89). Thus, short peptides (90), drugs (91), and natural compounds (92) were employed for inhibiting the Aβ aggregation and further inhibiting the cell toxicities. In our study, Ma-3-gal-Cl could bind with the Aβ monomer and fibrils (Figure 8), but the Ma-3-gal-Cl could bind with the N-terminal of the Aβ monomer (Figure 8), which could not inhibit Aβ aggregation at the early stage and resulted in the increase in ThT fluorescence intensity (Figure 6) and the formation of the aggregates (Figure 7) in the first 6 h. For the Aβ fibril, Ma-3-gal-Cl could bind with the U-shaped turn section of Aβ and formed pi–H interaction with Glu22, Phe19, Phe20, and Ala21 (Figure 8). These interactions of Ma-3-gal-Cl with Aβ fibril could inhibit the other Aβ aggregates (e.g., oligomers and protofibrils) and Aβ monomers to attach the formed Aβ fibrils and inhibit the further aggregation of Aβ. Thus, when the Aβ was incubated with Ma-3-gal-Cl after 6 h, the ThT fluorescence intensity was decreased (Figure 6) and the amount of Aβ aggregates was also decreased (Figure 7).

Neurodegenerative disease is a complex and multi-factorial disease, so the development of multi-target drugs for the prevention and delay of neurodegenerative disease is giving more hope (93–95). Therefore, this study attempts to explain the inhibition effect of Ma-3-gal-Cl on Aβ (1–42)/Cu^2+^/AA system resulting in SH-SY5Y cell apoptosis from the mechanism of antioxidation and inhibition of Aβ aggregation as follows: (1) Ma-3-gal-Cl could reduce the generation of intracellular ROS and maintain the mitochondrial membrane potential of Aβ (1–42)/Cu^2+^/AA treated cells *via* the decreased amount of hydroxyl radicals which produced by Aβ (1–42)/Cu^2+^/AA mixture. (2) Ma-3-gal-Cl could bind with the unactivated caspase-3 and the MKK6 to reduce the amount of activated caspase-3 and the phosphorylated p38 which induced by the Aβ (1–42)/Cu^2+^/AA system. (3) Ma-3-gal-Cl could bind with the Aβ monomer and fibrils to inhibit the Aβ aggregation (Figure 7).

## Conclusion

In this study, an effective Ma-3-gal-Cl was screened from bilberry with cheminformatics methods, and the activity and possible mechanism of the protective effect of Ma-3-gal-Cl to Aβ (1–42)/Cu^2+^/AA treated SH-SY5Y cells were investigated with *in vitro* experiments and *in silico* calculation. The experimental results showed that Ma-3-gal-Cl had effective protection for SH-SY5Y cells treated with Aβ (1–42)/Cu^2+^/AA by antioxidation and anti-Aβ aggregation effects. The molecular docking results provided detailed structure-based information for elucidating the multiple potential effects of Ma-3-gal-Cl against AD. Our findings suggested that Ma-3-gal-Cl could be a promising ingredient for drug development or dietary therapy for Alzheimer's disease.

## Data availability statement

The original contributions presented in the study are included in the article/Supplementary material, further inquiries can be directed to the corresponding author.

## Author contributions

RX: investigation, data curation, formal analysis, visualization, and writing—original draft. RL: visualization. Y-hC: validation. JD: software and writing—reviewing and editing. LZ: conceptualization, methodology, supervision, project administration, and funding acquisition. All authors contributed to the article and approved the submitted version.
