# Supplementary material for: Computational screening for new neuroprotective ingredients against Alzheimer's disease from bilberry by cheminformatics approaches
Source: Front Nutr. 2022 Dec 9;9:1061552. doi: 10.3389/fnut.2022.1061552 (PMC9780678; doi:10.3389/fnut.2022.1061552)

**Article title:**

**Computational screening for new neuroprotective ingredients against Alzheimer's disease from Bilberry by cheminformatics approaches**

**Authors:**

Ran Xiao ^a, c^, Rui Liang ^a^, Yunhui Cai ^a^, Jie Dong ^b^, and Lin Zhang *^, a^


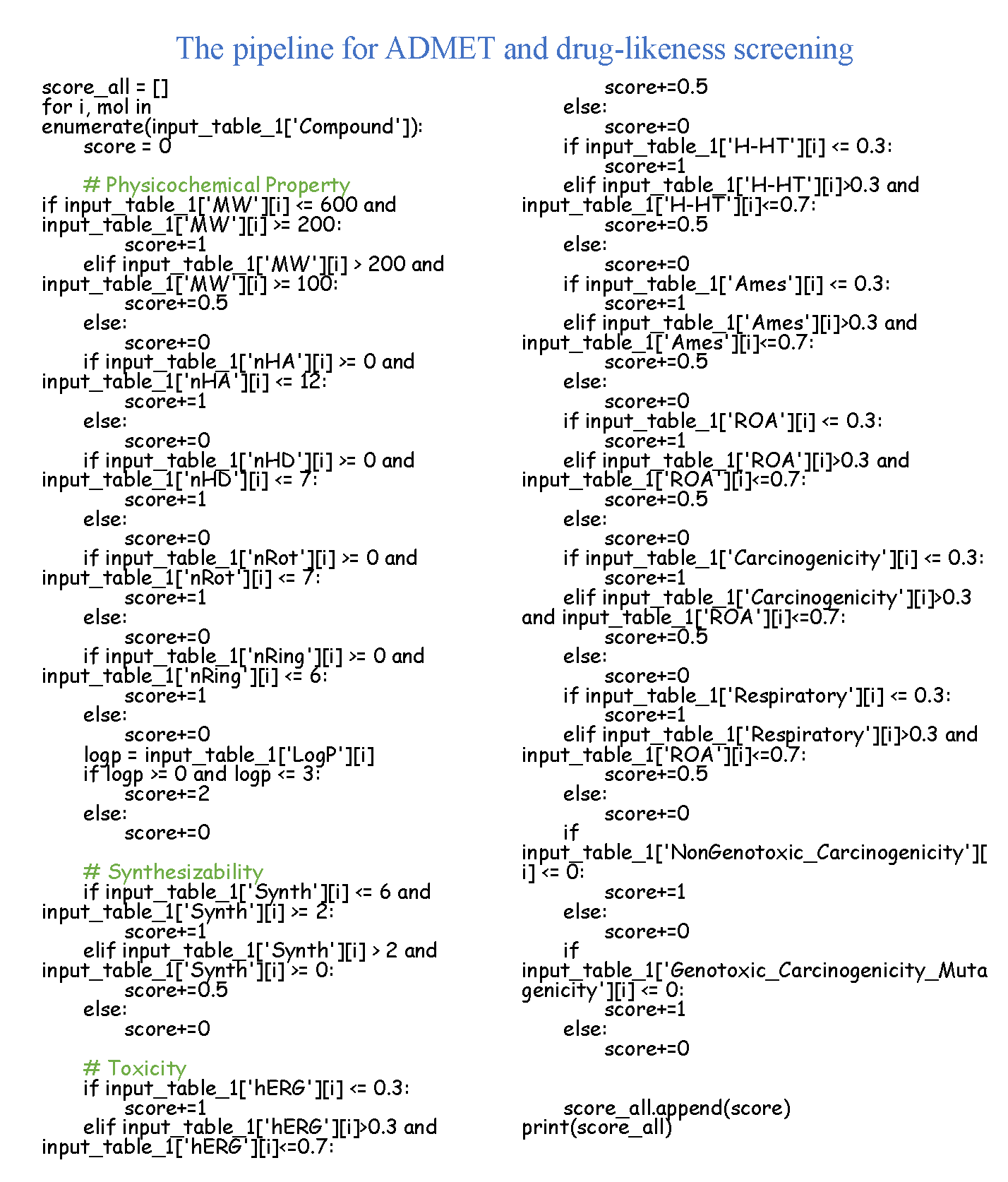


**Figure S1: the pipeline for the ADMET and Drug-likeness screening. The programming language was Python and the detailed definitions can be found at the website of ADMETlab and ADMETlab 2.0.**

To investigate the effect of Ma-3-gal-Cl on the cell viability of SH-SY5Y cells, the cell viabilities which incubated with different concentration of Ma-3-gal-Cl were detected as shown in Figure S1. When the concentration of Ma-3-gal-Cl was at 10 μmol/L, the cell viability was 98.0%. When the concentration of Ma-3-gal-Cl was increased, the cell viability was decreased. And when the concentration of Ma-3-gal-Cl was at 45 μmol/L, the cell viability was decreased to 72.1%.





**Figure S2. Cell viability of different concentration of Ma-3-gal-Cl treated SH-SY5Y cells. * p <0.05, * * p < 0.01 compared to control values.**

To investigate the effect of Aβ_(1-42)_, Cu^2+^, or ascobic acid (AA) on the cell viability of SH-SY5Y cells, the cell viabilities which incubated with these solutions were detected as shown in Figure S2. When the SH-SY5Y cells were incubated with Aβ_(1-42)_ (10 μmol/L) or Cu^2+^ (5 μmol/L) solutions, the cell viabilities were above 92.3%. And when the cells were incubated with AA (1 mmol/L), the cell viability was 74.5%. When the Aβ_(1-42)_/Cu^2+^/AA mixture treated with the cells, the cell viability was decreased to 55.0%.





**Figure S3. Cell viability of different Aβ, Cu^2+^, AA or the mixtures treated SH-SY5Y cells. * p <0.05, * * p < 0.01 compared to control values.**

**Table S1. The top 10 compounds kept after the ADMET and druglikeness screening.**


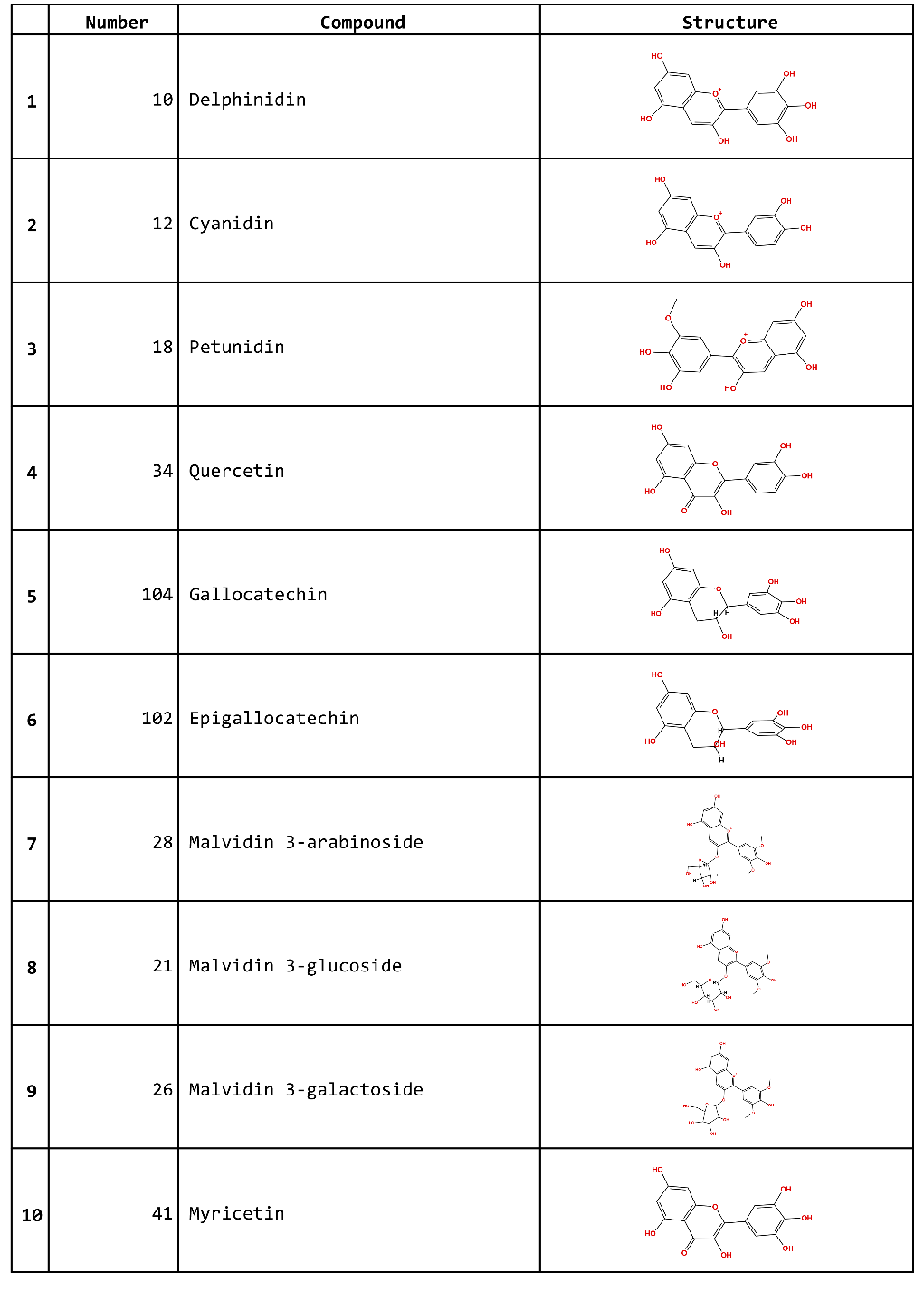

Supplement: Supplementary file 1 [file Data_Sheet_1.docx]
